# An Investigation into the Relationship between Owner Knowledge, Diet, and Dental Disease in Guinea Pigs (*Cavia porcellus*)

**DOI:** 10.3390/ani6110073

**Published:** 2016-11-14

**Authors:** Rosemary Norman, Alison P. Wills

**Affiliations:** Hartpury University Centre, Department of Animal and Land Science, Hartpury College, Hartpury, Gloucester, Gloucestershire GL19 3BE, UK; Rosemary-Eve.Harris@Hartpury.ac.uk

**Keywords:** guinea pigs, diet, dental disease, owner knowledge

## Abstract

**Simple Summary:**

Dental disease is a serious problem in small mammals, with cases in rabbits well documented. Guinea pigs also suffer from this condition, yet the literature investigating the underlying causes in guinea pigs is limited. Owners of guinea pigs were surveyed to investigate what they fed their animals. It was discovered that there was no relationship between the overall diet of the animals and whether or not they had been diagnosed with dental disease or displayed clinical signs of the disease. However, the environment was important, with animals that had access to the outside, including the use of runs on both concrete and grass, less likely to display clinical signs of disease. Some of the clinical signs of dental disease in guinea pigs, including difficulty eating, were related to dental problems. These findings are important, as many guinea pigs may not have continuous access to the outdoors. Dental disease is a serious welfare concern, as many owners may not pick up on the clinical signs, leaving animals susceptible to pain, dysphagia, malnutrition, and secondary infection. It is important that owners are aware of key clinical signs, particularly in multi-animal households where monitoring food consumption may be challenging.

**Abstract:**

Recent studies have highlighted a high prevalence of dental disease in domestic guinea pigs, yet the aetiology of this multi-factorial disease is still unclear. Factors that have been associated with dental disease include feeding a diet that is high in energy but low in fibre, feeding an insufficiently abrasive diet, a lack of dietary calcium, and genetics. As many of these factors relate to the husbandry requirements of guinea pigs, owner awareness of dietary requirements is of the utmost importance. An online questionnaire was created based on previous research into the husbandry and feeding of rabbits. Guinea pig owners were asked to answer questions on the clinical history of their animals and their diet and management. In total, 150 surveys were completed for 344 guinea pigs, where owners of multiple animals could complete the survey for individuals. According to the owners, 6.7% of guinea pigs had been clinically diagnosed with dental disease, but 16.6% had signs consistent with dental disease. The specific clinical signs of having difficulty eating (Exp(B) = 33.927, Nagelkerke R^2^ = 0.301, *p* < 0.05) and producing fewer or smaller faecal droppings (Exp(B) = 13.733, Nagelkerke R^2^ = 0.149, *p* < 0.05) were predictive for the presence of dental disease. Having access to an outside environment, including the use of runs on both concrete and grass, was significantly related to not displaying clinical signs of dental disease (Exp(B) = 1.894, Nagelkerke R^2^ = 0.021, *p* < 0.05). There was no significant relationship between owner knowledge, guinea pig diet, and dental disease in the study population. This study highlights the importance of access to the outdoors for the health and welfare of guinea pigs in addition to the need for owners to be alert to key clinical signs. A relationship between diet and dental disease was not identified in this study; however, the underlying aetiological causes of this condition require further investigation.

## 1. Introduction

The incisor and cheek teeth of small herbivorous mammals such as rabbits, guinea pigs, and chinchillas are open rooted and are continuously growing with open apices [1]. It has been suggested that this continual eruption of the teeth could be interpreted as an evolutionary adaptation to diets such as grasses, dust, and grit which possess both internal and external abrasiveness [2]. Guinea pig incisors have been reported to grow at 1.4–1.9 mm per week, with lower incisors growing 1.2–2.4 mm [3]. When these animals are subject to any process that interferes with the normal eruption of or wearing of their teeth, dental disease will occur [4].

The causes of dental disease can be divided into congenital and acquired [4]. Congenital factors leading to dental disease are seen commonly in pet rabbits [5]. However, these are considered relatively rare in rodents such as guinea pigs [3]. It is assumed that the majority of cases of dental disease in guinea pigs are acquired with aetiological hypotheses including trauma, abnormal dental wear resulting from an insufficient diet, and underlying metabolic bone disease due to a lack of calcium and vitamin D [6,7]. Trauma to the teeth can result from falls or chewing on improper caging and other non-food items. This can cause subsequent malocclusion, resulting in abnormal contact and insufficient wear during mastication, with resulting overgrowth of the teeth [8].

The most common clinical presentation seen in guinea pigs with dental disease is malocclusion of the cheek teeth (molars and premolars), with the mandibular teeth extending into the oral cavity in a characteristic ‘bridge’ formation thus trapping the tongue [9]. Incisor malocclusion can occur secondary to this, but rarely exists without either concurrent cheek teeth overgrowth or trauma [9]. Enamel spurs may form on the upper and lower cheek teeth either adjacent to the tongue or adjacent to the cheek, respectively. These can cause mucosal trauma and result in pain [10]. Elongation of the teeth will also result in extension of the apices of the reserve crowns into periapical tissue, which can lead to swelling. If the apices continue to extend into the ocular region, conjunctivitis or exophthalmos can develop [9]. Minarikova has suggested that acquired dental disease also predisposes guinea pigs to periodontal disease [11]. Dental disease can also occur secondarily to other illnesses in guinea pigs, which may induce anorexia. As guinea pigs possess hypsodont teeth, only a short period of decreased food consumption may be sufficient to induce dental disease.

Feeding a low fibre, high energy diet may result in a decreased volume of food being consumed, reducing the overall mastication time, thus reducing tooth wear [12,13]. This combined with a decrease in masticatory action from chewing on unnatural foods and lack of naturally abrasive food content can result in a 16-fold reduction in cheek tooth wear [14]. Whilst the lack of a sufficiently abrasive diet is a common aetiological hypothesis for the development of acquired dental disease in rabbits, the only study to experimentally investigate the effect of diet on tooth wear in guinea pigs did not find dietary abrasiveness to be significantly related to overgrowth of the teeth [3]. Therefore, further research is needed to elucidate the cause of dental disease in guinea pigs, since comparison to the research conducted in rabbits is not always beneficial due to their differing dental anatomy [15].

It has been theorised that a lack of calcium and vitamin D results in dental disease, and this is supported by a study in which intact skulls of pet rabbits were examined radiographically [16]. A positive correlation between the severity of dental disease and the frequency of recorded signs of osteopenia in the skull was identified, leading to the conclusion that loss of supporting bone, rather than increased tooth on tooth pressure, leads to extension of the tooth root. It has been suggested that rabbits’ diets may be lacking calcium and vitamin D because of the selective feeding behaviour of rabbits, in which rabbits naturally eat the components of the food high in energy and low in fibre [17]. Whilst it is accepted that guinea pigs also require a source of vitamin D, whether vitamin D deficiency can cause dental disease in guinea pigs has yet to be investigated [7].

In recent years, the amount of research into owner awareness of the husbandry requirements of rabbits has rapidly increased, perhaps due to their increasing popularity as a companion animal and increased awareness of their welfare. One study examined 102 pet rabbits and found several health and welfare problems including dental disease, where the majority of owners of rabbits with signs of dental disease were not aware of the problem [18]. Other authors have investigated the knowledge and attitude of pet rabbit owners at the point of sale and found that respondents had a limited knowledge of the husbandry of rabbits, particularly in relation to their dietary needs [19,20].

Similar studies for guinea pigs are lacking even though there is a high prevalence of dental disease among guinea pigs [21]. In recent clinical studies, dental disease in guinea pigs was diagnosed in 12% to 23.4% of the animals, respectively [22,23], with one study identifying dental disease in 36.3% of the animals presented to the veterinary practice [21]. Poor husbandry and diet have been recognised as the leading cause of morbidity and mortality in guinea pigs, and clinical signs of illness shown by guinea pigs are often overlooked by owners [24]. Clinical signs associated with dental disease in guinea pigs include reduced appetite, weight loss, difficulty chewing, hypersalivation (also referred to as ‘slobbers’), decreased grooming, facial swelling, and an inability to perform coprophagy [25,26,27]. A recent article highlighted the need for further research into guinea pig medicine, emphasising the need for both owner education and preventative interventions to improve guinea pig welfare [28].

The aim of this study was to investigate guinea pig owners’ understanding of the health and husbandry of guinea pigs and to identify whether a relationship exists between owner knowledge and the presence of dental disease (or clinical signs thereof) in guinea pigs. It was also investigated whether there is a relationship between feeding guinea pigs a diet high in energy and low in fibre and the presence of dental disease. Finally, this study aimed to discover whether there is a relationship between feeding diets low in calcium and vitamin D, and dental disease, as has been demonstrated in rabbits [17].

## 2. Materials and Methods

### 2.1. Questionnaire Design and Distribution

A questionnaire based on previous studies investigating owner knowledge of the husbandry requirements of rabbits [18,19,29] was created for the purposes of this study. The questionnaire consisted of 48 questions in total, with a combination of closed, open, ranked, and multiple choice responses. The questionnaire was promoted online on guinea pig pages via the social media website Facebook™, with respondents identified via convenience sampling. The questionnaire was also publicly posted online on guinea pig forums. It was available for three months from January through to March 2016. The questionnaire was targeted at individuals over the age of eighteen who either currently or previously had owned or cared for guinea pigs. All participants contributed voluntarily, with no identifying personal data collected and all responses securely stored. All participants provided informed consent before completing the questionnaire. Owners were asked to provide demographic information and details of their guinea pig(s), guinea pig(s) diet and husbandry, any clinical history of dental disease, and any observed clinical signs of dental disease identified in their guinea pigs. For animals that did have a clinical history of dental disease, respondents were able to select the specific diagnosis (for example, malocclusion). Respondents were asked whether their animal(s) had received a veterinary diagnosis of dental disease which was intended to reduce the possibility of owner or lay person diagnoses being included. Respondents were able to complete the questionnaire for up to five individual guinea pigs.

### 2.2. Guinea Pig Health, Husbandry, and Diet

Open, ranked, and multiple choice questions about the guinea pig’s diet were posed in order to gain detailed information on what animals were fed. All specific individual dietary components were recorded for each guinea pig. In addition, a diet score was generated for each animal based on the levels of calcium, vitamin D, and fibre identified in the components of the guinea pig’s diet. Guinea pigs fed food high in each of these variables were awarded a higher diet score compared to guinea pigs fed foodstuff lacking in these components. For Vitamin D, animals given access to an outdoor environment scored higher than those kept indoors, to generate a rating for vitamin D based on both diet and ultraviolet (UV) light exposure [29]. In addition to the diet score, all individual dietary and environmental variables were analysed (Table 1).

### 2.3. Owner Knowledge

Statements about the husbandry, health, and welfare of guinea pigs were posed, and respondents were asked to rate their level of agreement with a Likert style statement. Owner responses were used to produce an overall knowledge score for each respondent. Each statement scored between 0 and 5 points; with maximum points allocated for strongly agreeing with a correct statement and no points allocated for strongly disagreeing with a correct statement. Incorrect statements were reverse coded. Respondents scored higher for submitting an answer of ‘neither agree nor disagree’ in comparison to getting an answer wrong.

The respondents were also asked to rate their perceived knowledge of dental disease in guinea pigs, on a scale between poor and excellent. In addition, respondents were given the opportunity to report any educational information about guinea pigs they had regular access to.

### 2.4. Statistical Analysis

All data were analysed using SPSS (version 22.0, SPSS Inc., Armonk, NY, USA) with a significance level of *p* < 0.05 applied. Data were tested for normality with the Kolmogorov-Smirnoff test. A Spearman’s Ranked Correlation was used to determine whether there was a relationship between how respondents rated their knowledge and their actual knowledge score.

Binary Logistic Regression was performed to assess whether any aspects of the husbandry and diet of the guinea pigs were predictive for the presence of dental disease or clinical signs of dental disease. This model had a number of variables (Table 1); the type of concentrated food the guinea pig was fed, any vitamin supplements it was given, whether hay was given, and, if so, what type of hay and how it was given, whether the guinea pig was given fresh food, and, if so, how often, any other treats given, and whether the guinea pig had access to the sun (for vitamin D). In addition, the overall diet score was analysed, as was the owner knowledge score and perceived owner knowledge, to identify whether these were predictive for the presence of dental disease or clinical signs of dental disease in guinea pigs.

There is a calculation to determine the minimum sample size for logistic regression to ensure accuracy of results; this was calculated for this study as *n* = 149 [30]. The sample size for this study was 344, therefore the results were valid. The knowledge score data were non-parametric; however, both the binary logistic regression and Spearman’s Ranked correlation test do not assume normality, so the results were viable [31].

## 3. Results

There were 150 online surveys completed, for a total of 344 guinea pigs. We found that 94% of respondents were female, and the majority were in the 22–30 (31.3%) or 41–50 (24%) age group. We found that 94.7% of respondents were the main care provider for their guinea pig(s). The most frequently reported age of a guinea pig was 2 to 3 years old, 56.7% of guinea pigs were female, 42.7% were male, and for 0.6% the sex was not known. In the sample population, 42.2% of guinea pigs had access to a run outside, and 6.7% of guinea pigs were kept constantly in their hutch.

### 3.1. Diet

The majority of guinea pigs (77.9%) were fed pellets as their staple food, 11.3% were fed a mix of pellets and muesli, 9.6% were fed muesli, and 1.2% were fed another form of staple food. Of those surveyed, 25.3% of owners gave their guinea pig some form of vitamin supplement, with the most popular vitamin supplement being vitamin C (24.1%). Almost all guinea pigs (99.4%) were fed hay, with Timothy and Meadow hay being the most popular type (44.2% and 42.7%, respectively). Putting hay in both a hay rack and loose in the hutch was the most common way to feed hay (41%). However, 40.1% of owners just fed hay loose in the hutch, 15.4% of owners just fed hay in a hay rack, and 3.5% of owners fed hay in a different way. Ninety eight percent of guinea pigs were fed a range of fresh food, with 7.3% being fed fresh food more than twice a day, 44.2% twice a day, 40.1% everyday, 4.1% every other day, 2.3% less than every other day, and 2% were not given fresh food. Some guinea pigs were also fed cut grass (47.4%), and 21.5% of guinea pigs were fed treats. There was no significant relationship between any specific individual dietary components and the presence of dental disease.

Guinea pigs given vitamin supplements were more likely to have dental disease than a guinea pig not given vitamin supplements (Exp(B) = 2.955, Nagelkerke R^2^ = 0.043, *p* < 0.05). Giving guinea pigs a vitamin C supplement specifically was related to an increased likelihood of dental disease being present (Exp(B) = 2.613, Nagelkerke R^2^ = 0.033, *p* < 0.05). There was no significant relationship found between a low overall diet score (feeding diets low in calcium, vitamin D, and fibre) and the presence of dental disease.

### 3.2. Dental Disease and Clinical Signs

We found that 6.7% of guinea pigs had previously been diagnosed with dental disease by a veterinarian, with the disorder being related to overgrown cheek teeth in 2.6% of cases, overgrown incisors in 1.2% of cases, tooth root abscesses in 1.5% of cases, broken teeth in 0.9% of cases, and malocclusion in 0.6% of cases. However, 16.6% of guinea pigs displayed one or more clinical signs consistent with dental disease.

A guinea pig that displayed clinical signs was more likely (Exp(B) = 26.031, Nagelkerke R^2^ = 0.332, *p* < 0.05) to have a veterinary confirmed diagnosis of dental disease than a guinea pig that was not displaying any clinical signs. The specific clinical signs of having difficulty eating (Exp(B) = 33.927, Nagelkerke R^2^ = 0.301, *p* < 0.05) and producing fewer or smaller faecal droppings (Exp(B) = 13.733, Nagelkerke R^2^ = 0.149, *p* < 0.05) were significantly related to the presence of dental disease. Having access to an outside environment was significantly related to not displaying clinical signs of dental disease (Exp(B) = 1.894, Nagelkerke R^2^ = 0.021, *p* < 0.05).

### 3.3. Knowledge Scores

Out of a maximum score of 100, the mean knowledge rating was 52.50, and the mean actual knowledge score was 73.83. There was a positive correlation (R = 0.282) between actual knowledge score and knowledge rating (*p* < 0.05). Furthermore, owners who rated their knowledge highly were more likely to have a guinea pig with dental disease (Exp(B) = 1.033, Nagelkerke R^2^ = 0.099, *p* < 0.05).

## 4. Discussion

### 4.1. Diet

Guinea pigs originate from mountainous areas of South America, where the vegetation is tough, fibrous, and contains soil dust. Therefore, it has been suggested that highly abrasive food, of little nutritional value should be eaten in large quantities to encourage correct dental wear [32]. However, guinea pigs in captivity often receive concentrates in the form of pellets or muesli, and may have limited access to outdoor grazing [9].

This study found no relationship between animals being fed commercial diets of muesli, which is high in energy and low in fibrous content [6], and the presence of dental disease. There was also no relationship found between animals fed a lack of food with naturally abrasive content, such as hay and grass, and dental disease. However, in this study only 9.6% of guinea pigs were fed muesli and 99.4% of guinea pigs were fed hay, making it challenging to be able to rule out these factors as a cause of dental disease in guinea pigs. The lack of a relationship between an abrasive diet and dental disease is consistent with a previous study investigating the effect of diet on tooth wear in guinea pigs [3]. Furthermore, guinea pigs with access to the outdoors were significantly less likely to display clinical signs of dental disease. This may suggest that access to natural grazing may promote more effective dental wear than unlimited access to hay which 99.4% of owners provided.

### 4.2. Environmental Factors

Metabolic bone disease is a progressive syndrome that affects the shape, position, and structure of the teeth, due to a lack of dietary calcium and vitamin D, and has been well documented in rabbits [3,6]. Vitamin D has a direct effect on the mineralisation of bones and teeth [7]. There was no significant relationship found between guinea pigs being fed diets low in vitamin D, calcium, and fibre and dental disease in this study. However, having access to an outside environment was found to be significantly related to guinea pigs not displaying clinical signs of dental disease. This might suggest that access to vitamin D from sunlight is related to the presence of clinical signs of dental disease, or dental disease itself in guinea pigs. A previous study found that rabbits kept in hutches without access to sunlight, had low or undetectable plasma concentrations of vitamin D, whereas rabbits which had increased access to sunlight had higher concentrations of vitamin D [29]. However, this study did not measure serum concentrations of vitamin D; hence it is unclear what aspect of the outdoor environment is protective against the development of dental disease in guinea pigs and this warrants further research.

### 4.3. Vitamin C

Almost one quarter of the guinea pigs (24.1%) received vitamin C supplements. The most likely explanation for this finding would be that owners were aware that guinea pigs cannot synthesise their own vitamin C [3]. However, there was a significant relationship found between guinea pigs being fed vitamin C supplements and the presence of dental disease in this study. This was a surprising finding, but one explanation would be that knowledgeable owners are more likely to both supplement their guinea pigs’ diet and be able to detect signs of dental disease in their pets. An alternative explanation would be that owners tend to supplement ill animals due to their higher vitamin C requirements.

Williams and Sullivan [33] found that an excess of vitamin C can cause ocular abnormalities in guinea pigs, but the risks associated with hypovitaminosis C appear to outweigh any adverse effects associated with excessive supplementation. However, a high percentage of owners supplementing their animals with vitamin C was also seen in a similar study with the authors stating that the management of dietary uptake of vitamin C in guinea pigs should always be discussed [21]. Why many owners decide to supplement their animals in addition to providing pelleted foods that contain vitamin C and feeding fresh vegetables high in vitamin C is unclear.

### 4.4. Dental Disease

This study only found 6.7% of the guinea pigs to have been clinically diagnosed with dental disease, which is low in comparison to other studies [8,22]. However, due to data being collected in the form of a questionnaire, the clinical diagnosis of dental disease was reported by respondents, so it could be hypothesised that some guinea pigs had dental disease even though a clinical diagnosis had not been made. This is likely, as clinical signs in guinea pigs are often overlooked by owners [24]. Guinea pigs were also not physically examined by the experimenter, as was performed in previous studies [21,34,35]. Additionally, as dental disease can be difficult to diagnose due to guinea pigs typically having a small mouth opening and a long narrow oral cavity, the true prevalence is often underestimated even with a clinical examination [5]. An otoscope can be used to aid in visual inspection of the oral cavity in the conscious animal, but most authors suggest that general anaesthetic is required for a definitive diagnosis [15].

Clinical signs of dental disease in this study were reported in 16.6% of guinea pigs. A significant relationship was found between guinea pigs that displayed clinical signs associated with dental disease and the presence of dental disease itself. In particular, the clinical signs of difficulty eating and producing fewer or smaller faecal pellets were significantly related to the presence of dental disease. This is an important finding, as many clinical signs are subtle, and owners may require further education on what to look for in their pets.

### 4.5. Owner Knowledge

In general, respondents achieved a high knowledge score, with a mean knowledge score of 73.83. However, respondents in this study were not representative of guinea pig owners as a whole due to both the sample size and the method of obtaining participants. It could be assumed that owners who actively participate in online groups and forums might be more informed on and invested in the health and husbandry of their animals than those that do not belong to an online group.

A positive correlation was found between respondents’ perceived knowledge and their actual knowledge scores. A relatively high proportion of respondents did not realise that malocclusion and traumatic injuries were common causes of dental disease, which was in contrast to the generally high knowledge scores seen. This could be explained by respondents believing that genetic factors can play a role in dental disease. Whilst congenital causes are accepted in rabbits, they are generally believed to occur less frequently in guinea pigs and other rodents [3]. Respondents were presented with a picture in the questionnaire of a guinea pig with very obvious elongation of the incisors; however, 17.4% of respondents did not identify this animal as having dental disease. This is similar to a study by Mullan and Main [18], which found owners unable to identify dental disease in pet rabbits. In this study, owners who rated their knowledge highly were more likely to have a guinea pig with dental disease. This may again relate to the ability of knowledgeable owners to identify disease in their animals.

## 5. Conclusions

In conclusion, 6.7% of owners reported their guinea pigs to have been diagnosed with dental disease by a veterinarian. Owners were generally well educated and informed on the husbandry and dietary requirements of guinea pigs. This contradicts some authors that state that the high prevalence of dental disease observed in some studies is a result of owners failing to feed a high fibre diet. In rabbits, muesli style commercial foods have been implicated in the development of dental disease, but in this study no relationship was found in guinea pigs between diet and dental disease. However, this study does highlight the importance of owners noticing key clinical signs such as decreased food consumption. Access to the outdoors was identified as important in maintaining dental health; hence the role of vitamin D in acquired dental disease in guinea pigs requires further investigation.

## Figures and Tables

**Table 1:** Variables related to guinea pig diet and husbandry that were included in the binary logistic regression model.

Independent Variables	Dependent Variables	
	Variable Type	Variable
Clinical signs	Concentrated food	Muesli
Dental disease		Pellets
		Mix of muesli and pellets
		Other
		None
	Hay type	Meadow hay
		Timothy hay
		Herbal hay
		Orchard hay
		Oat hay
		Other
		None
	Provision of Hay	Loose in hutch
		In a hay rack
		Both loose and in a hay rack
		Other
		None given
	Type of fresh food fed	Vegetables
		Salad leaves
		Fruit
		Garden plants
		Other
		None
	Frequency of provision of fresh food	More than twice a day
		Twice a day
		Every day
		Every other day
		Less than every other day
		None
	Other food(s)	Cut grass
		Breakfast cereal
		Guinea pig treats
		Other
		None
	Vitamin supplements	Vitamin C
		Vitamin D
		Vitamin B
		Calcium
		Fluids and electrolytes
		Other
		None
	Environmental	Kept indoors
		Kept outside in a hutch
		Kept outside in a run
		Kept outside in a garden
	Diet score	Diet score
	Owner knowledge	Perceived knowledge
		Actual knowledge
	Guinea pig	Age
		Sex
		Weight
		Breed
